# Characteristics Influencing Adherence to SARS-CoV-2 Serial Antigen Testing Following a Federal Safety Communication: Cohort Study

**DOI:** 10.2196/92002

**Published:** 2026-07-15

**Authors:** Micajah Z McGarity, Catherine C Pollack, Elizabeth A Currier, Andrew C Godoshian, Zachary R Smith, Marcus Siddall, Gregg M Tavolacci, Reynolds M Salerno, Thomas C Tsai, Michael F Iademarco, Joseph D Miller

**Affiliations:** 1 Johns Hopkins University Applied Physics Laboratory Laurel, MD United States; 2 Increasing Community Access to Testing, Treatment, and Response Program Centers for Disease Control and Prevention Atlanta, GA United States; 3 Walgreens Co Deerfield, IL United States; 4 eTrueNorth Fort Worth, TX United States; 5 Office of Laboratory Systems and Response Centers for Disease Control and Prevention Atlanta, GA United States; 6 Department of Health Policy and Management Harvard T.H. Chan School of Public Health Harvard University Boston, MA United States; 7 (retired) United States Public Health Service Washington, DC United States

**Keywords:** antigen testing, at-home testing, Centers for Disease Control and Prevention, community testing, COVID-19, diagnostic testing strategy, false-negative, public health surveillance, real-world evidence, repeat testing, risk communication, serial testing, testing adherence

## Abstract

**Background:**

Throughout the COVID-19 pandemic, regular population testing for SARS-CoV-2 was crucial for disease monitoring and management. Initially, reverse transcriptase–polymerase chain reaction tests were primarily used for identifying new cases, but their high cost and delayed results, combined with laboratory capacity and supply chain limitations, led to the adoption of antigen-detection rapid diagnostic tests (Ag-RDTs) at point-of-care locations. On August 11, 2022, the US Food and Drug Administration (FDA) issued a safety communication recommending serial Ag-RDT testing to reduce the risk of false-negative results from singular testing and combat disease spread, followed by a revision letter on November 1, 2022, to manufacturers to update their authorized product labeling.

**Objective:**

This study evaluates adherence to serial testing using data from the Centers for Disease Control and Prevention (CDC) Increasing Community Access to Testing, Treatment, and Response (ICATT) program.

**Methods:**

This national, retrospective cohort study analyzed ICATT program line-level testing records from August 12, 2022, to January 14, 2025. Patients were included if they had at least 1 Ag-RDT. Adherence was determined based on whether patients followed FDA guidance after a negative test. Multivariable logistic regression models were used to identify demographic features associated with adherence, with an expanded model to identify the impact of additional clinical variables. Rates of testing positive after an initial negative Ag-RDT were also calculated for ICATT pharmacy testing.

**Results:**

There were 2,189,464 patients included in the second-test adherence analysis, and 6813 included in the third-test adherence analysis. Less than 1% (20,290/2,189,464) of patients were adherent to the instructions to use a second Ag-RDT, and 98.2% (2,149,292/2,189,464) had no second test documented in ICATT and therefore were classified as nonadherent in this analysis. Second-test adherence was higher among Asian (adjusted odds ratio [AOR] 1.28, 95% CI 1.21-1.36) and Black (AOR 1.17, 95% CI 1.12-1.23) patients and lower among children (AOR 0.48, 95% CI 0.45-0.51). In the expanded model, recent COVID-19 contact increased adherence odds (AOR 1.87, 95% CI 1.64-2.13), whereas recent COVID-19 infection decreased them (AOR 0.66, 95% CI 0.56-0.76). Among patients with symptoms initially testing negative with an Ag-RDT, 24.9% (7310/29,315) tested positive on their second test, and 57.4% (4195/7310) of these episodes were adherent. For patients without symptoms, 4.2% (60/1435) tested positive on their third test after testing negative on their first 2 tests, with 60% (36/60) adherence.

**Conclusions:**

This study is the first to evaluate adherence to the FDA instructions to perform serial Ag-RDTs. Overall, adherence was low among patients seeking testing at community sites. Recent COVID-19 exposure was associated with the highest odds of serial testing adherence. These insights can inform targeted public health strategies to improve adherence and reduce the risk of false-negative results from Ag-RDTs in future pandemics.

## Introduction

Throughout the COVID-19 pandemic, regular population testing for SARS-CoV-2 was a cornerstone of disease monitoring and management. Early in the pandemic, nucleic acid amplification tests (NAATs), which include reverse transcriptase–polymerase chain reaction (RT-PCR) tests, were relied upon as the sole way to identify new cases because they were the first diagnostic tests developed and authorized by the US Food and Drug Administration (FDA). While highly sensitive, RT-PCR tests were expensive, and results may not be available for several days due to shipping delays, staffing shortages, supply chain shortages, and reporting backlogs [1,2]. The emergence of antigen-detection rapid diagnostic tests (Ag-RDTs) improved the accessibility and timeliness of SARS-CoV-2 testing, and these tests were less sensitive than their NAAT counterparts [3].

On August 11, 2022, the US FDA issued a new safety communication for serial Ag-RDT testing for SARS-CoV-2 to reduce the risk of false-negative results for COVID-19 [4-6]. The safety communication instructed individuals with COVID-19–like symptoms who received negative test results to test 48 hours after the first test. Asymptomatic individuals with a negative result were instructed to test 48 hours after the first test and then again after 48 hours if the first repeat test was also negative. Confirmatory “laboratory molecular-based” testing, including RT-PCR, was also encouraged, when possible, to further reduce the risk of false-negative Ag-RDT results. For both symptomatic and asymptomatic individuals, a positive result in any repeat test was considered indicative of a case of COVID-19. A comprehensive study of more than 7000 participants was used to establish these instructions and determined that serial Ag-RDT testing improved sensitivity for both Delta and Omicron SARS-CoV-2 variants [7,8].

The degree to which the US population followed the FDA’s instructions has not been studied, primarily because most Ag-RDTs were performed at home, making studies of Ag-RDT testing more difficult to conduct. While it was possible for patients to voluntarily report at-home test results for 4 Ag-RDT manufacturers, that reporting was only a fraction of the total testing volume, and most results were self-interpreted and self-reported [9]. This was partially rectified by the MakeMyTestCount program launched by the US National Institutes of Health’s Rapid Acceleration of Diagnostics Tech program in December 2022 [10]. However, there have been no studies to date that have evaluated adherence to serial Ag-RDT testing within that program. Studies that have evaluated serial test adherence have either been outside the United States, within a closed cohort, or have been small, limiting understanding of how the specific FDA instructions generalize to a broader US population [11-15]. Understanding whether COVID-19 testing among the public adhered to the authorized instructions for use of antigen tests would provide important information for public health officials and epidemiologists to optimize diagnostic testing for future respiratory outbreaks.

The US Centers for Disease Control and Prevention (CDC) Increasing Community Access to Testing, Treatment, and Response (ICATT) program began in April 2020 to provide no-cost SARS-CoV-2 testing through a public-private partnership with participating pharmacy vendors, including CVS, Rite Aid, Walgreens, and eTrueNorth, among others [16]. Since its creation, the ICATT program has collected data on 61 million SARS-CoV-2 tests at more than 19,000 locations in all 50 states, Puerto Rico, and Washington DC. Of the 61 million tests, 49.4 million were categorized as “ICATT paid” or “ICATT conducted,” and 12 million were categorized as “non-ICATT test” records performed at ICATT locations. Thus, the ICATT dataset captures testing that occurred at participating vendor sites and does not capture at-home testing or testing performed at non-ICATT sites. In July 2022, ICATT program testing vendors began assigning unique patient identification numbers to individual testing records, allowing for a large-scale, longitudinal study of adherence to FDA serial testing instructions. In addition, the ICATT dataset included symptom status, demographic information, and clinical features for each testing record to study factors that affect adherence. This study aims to identify patient-level demographic and clinical features associated with adherence to FDA serial Ag-RDT testing guidelines within a national retrospective cohort.

## Methods

### Data and Key Variables

ICATT-funded line-level testing records were used as the primary source of data (49.4 million), in addition to Medicare, Medicaid, private insurers, Health Resources and Services Administration (HRSA), and cash-paid pharmacy test records (11.9 million) at ICATT locations. These records reflect on-site testing at participating ICATT vendor locations and therefore do not include at-home tests or testing performed at non-ICATT sites. ICATT testing records were provided by ICATT testing sites to CDC as part of CDC’s One CDC Data Platform (1CDP). Individual testing records were combined into testing events (testing records from the same patient and the same vendor on the same day). Testing events that occurred within 10 days of each other were collapsed into testing episodes via daisy-chaining to link distinct testing events for each patient. Adherence with serial testing was then determined for each testing episode. Finally, summaries of adherence were created for each patient, demographic and clinical features were joined to patient records, and inclusion/exclusion criteria were applied.

**Figure 1:**
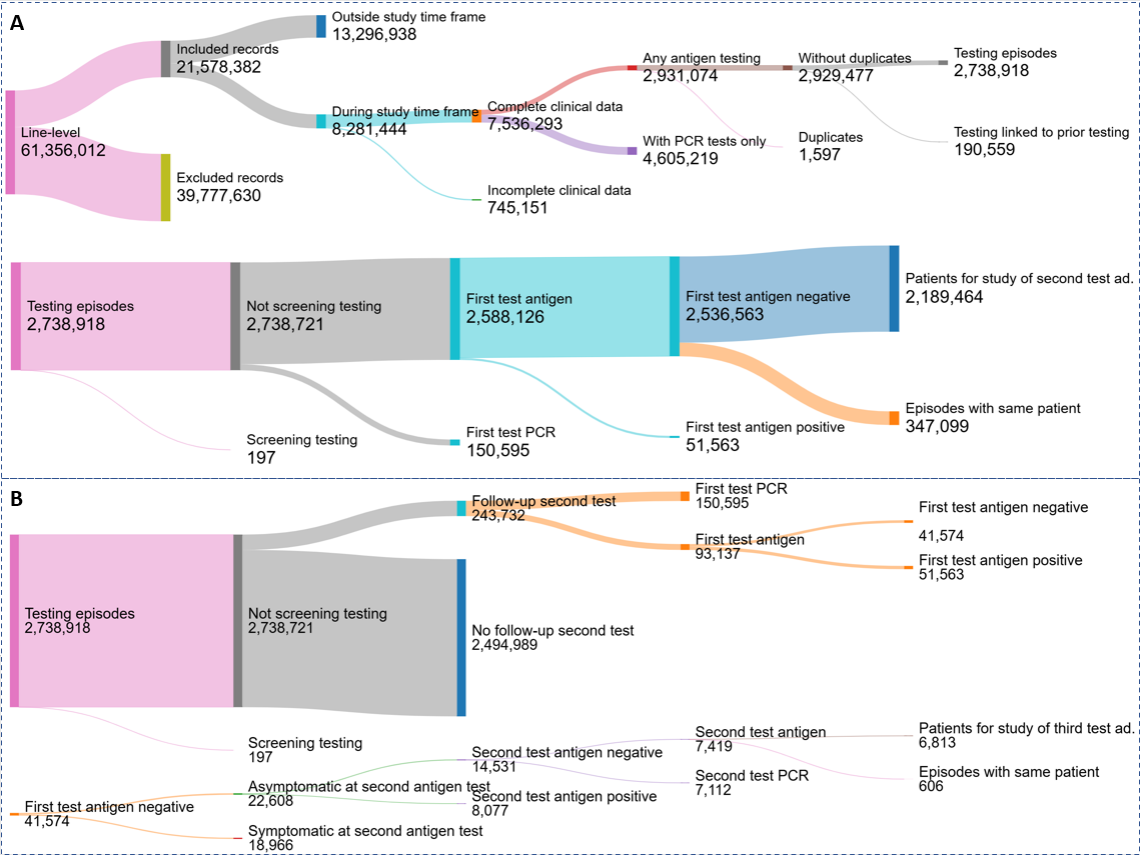
Tests-to-patients flow diagram for second-test and third-test adherence analyses. (A) Flow for the second-test adherence analysis; (B) Flow for the third-test adherence analysis. The term polymerase chain reaction (PCR) is used in the figure to denote any nucleic acid amplification test (NAAT).

There were 745,151 testing records excluded because the symptom status was unreported, and it was used to determine whether a patient was required to take 2 or 3 Ag-RDTs according to the authorized instructions for use. Patients were included if they had at least 1 Ag-RDT between August 12, 2022, and January 14, 2025, at a CVS, Walgreens, or eTrueNorth facility. If the duration of a patient’s testing episode exceeded 30 days and the number of tests per day exceeded 0.2, the episode was excluded from the study (197 episodes), as this was considered to be indicative of regular screening testing and not diagnostic testing for acute illness. Since the primary outcome of interest was adherence with FDA serial Ag-RDT testing instructions after a negative first test, patients were also excluded if their first test was a NAAT/RT-PCR test (150,595 episodes) or if their first test result was positive (51,563 episodes). In addition, 347,099 testing episodes were merged with other episodes with the same patient ID, and averages were calculated for each patient’s numeric or binary variables, leaving 2,189,464 patients eligible for second-test adherence analysis. The number of tests included and excluded based on different criteria is shown in Figure 1.

In an expanded model, available self-reported clinical variables, including recent contact with a patient infected with COVID-19, vaccination status, COVID-19 infection within the past 90 days, and symptom status at the time of testing, were examined alongside demographic features including age, sex, race, ethnicity, insurance status, test vendor (eg, CVS or Walgreens), and location (collapsed into US Census regions). To account for multiple testing episodes by the same patient, demographic variables for each testing episode were assigned based on the demographic information available for the first test of the testing episode. Clinical variables were represented as the rate of episodes in which that clinical variable occurred (eg, the proportion of episodes where the patient was symptomatic).

### Patient Summary Adherence Determination

**Figure 2:**
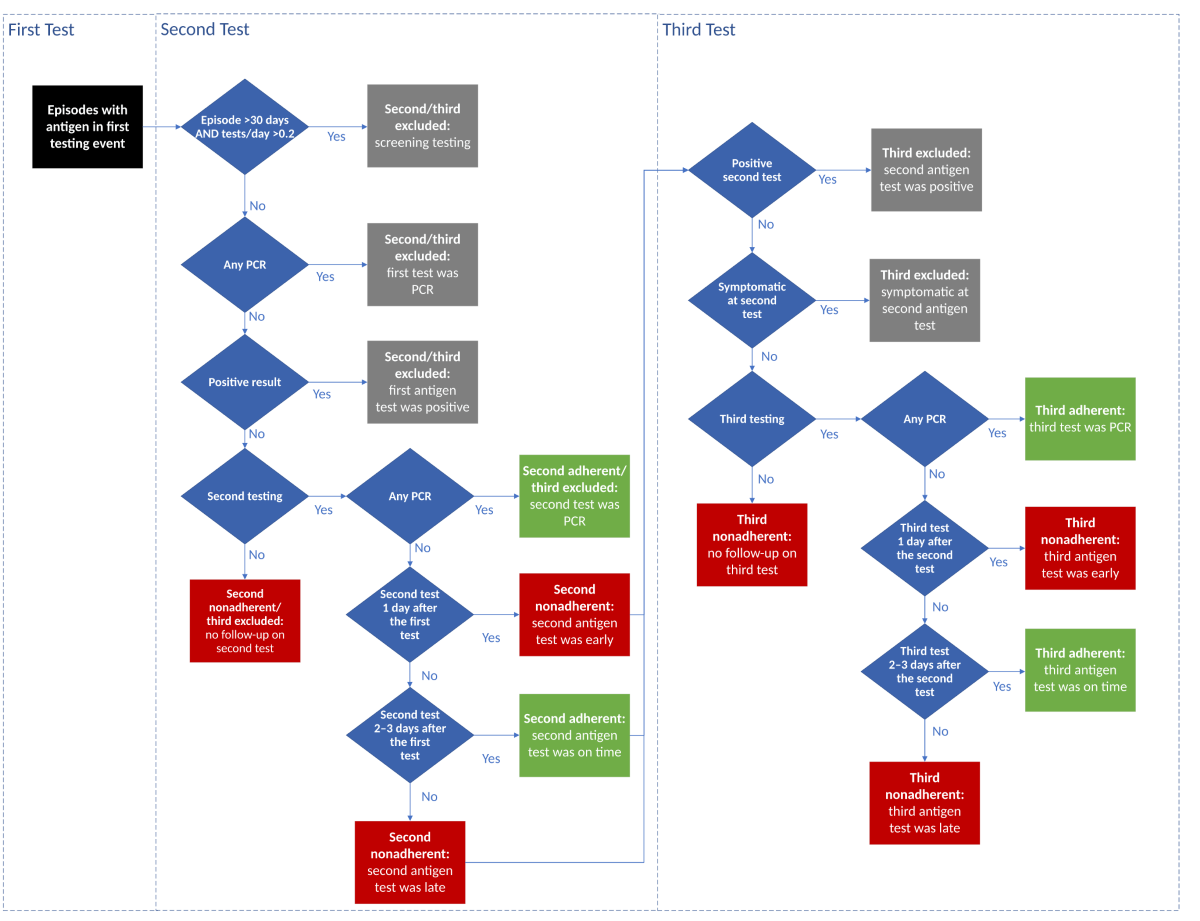
Serial testing adherence logic flowchart. The term polymerase chain reaction (PCR) is used in the figure to denote any nucleic acid amplification test (NAAT). Testing results were collapsed into episodes by grouping patient tests that occurred within 10 days of each other. Testing episodes exceeding both 30 days and 0.2 tests per day were excluded. After the initial test, testing was considered second-test adherent or third-test adherent based on the FDA safety communication [4]. Blue diamonds represent decision points, gray boxes indicate excluded testing episodes, red boxes indicate nonadherent episodes, and green boxes indicate adherent episodes.

Adherence was defined at the testing-episode level. A testing episode included all tests for the same patient that occurred within 10 days of one another. Episodes were excluded if they lasted more than 30 days and reflected frequent screening (>0.2 tests per day), began with a NAAT/RT-PCR test, or began with a positive Ag-RDT result. A series of complex branching logic (Figure 2) was then applied to the episodes to determine adherence with serial testing.

For second-test adherence, the first eligible test had to be a negative Ag-RDT. An episode was classified as second-test adherent if the next documented ICATT test was either (1) a NAAT/RT-PCR test or (2) a second Ag-RDT performed 2-3 days after the first negative Ag-RDT. An episode was classified as second-test nonadherent if no follow-up test was documented in ICATT, if the second Ag-RDT was performed 1 day after the first test, or if it was performed 4 or more days later.

For third-test adherence, only episodes in which the patient was asymptomatic and the second Ag-RDT was negative were eligible. These episodes were classified as third-test adherent if the next documented ICATT test was either (1) a NAAT/RT-PCR test or (2) a third Ag-RDT performed 2-3 days after the second negative Ag-RDT. They were classified as third-test nonadherent if no third test was documented in ICATT, if the third Ag-RDT was performed 1 day after the second test, or if it was performed 4 or more days later. Episodes with patients with symptoms or a positive second test were excluded from the third-test analysis because a third test was not recommended under FDA instructions [4]. For example, a patient without symptoms with negative Ag-RDTs on day 0, day 2, and day 4 would be classified as adherent for both the second and third tests; if the second test occurred on day 1 or day 4, or if no follow-up test was documented in ICATT, the episode would be classified as nonadherent.

Because some patients contributed more than 1 eligible testing episode, second-test adherence and third-test adherence were summarized at the patient level as the proportion of that patient’s eligible episodes that were adherent. This approach was used to avoid overweighting patients with repeated episodes and to reduce bias that could arise from treating correlated episodes from the same individual as independent observations. Although this aggregation may smooth within-patient variability across episodes, it allowed each patient to contribute a single outcome value to the regression analyses. For example, a patient with 2 adherent testing episodes and 1 nonadherent testing episode was considered 66.67% adherent.

### Statistical Modeling

Two separate generalized linear models with a binomial family and logit link function were fit to model the second- and third-test Ag-RDT serial testing adherence. For each model, the outcome variable was the patient-level proportion of eligible testing episodes that were adherent. A patient-level analytic approach was selected because some individuals contributed multiple testing episodes; analyzing episodes as independent observations could disproportionately weight frequent testers and underestimate within-patient correlation. All demographic variables and symptom status were included in the model as candidate predictors. To understand whether the time since the initial safety communication affected adherence, we also included calendar quarters since the initial announcement in the model. In an expanded model, the remaining 3 clinical variables were included in the model; these were included separately due to the high degree of missingness to avoid skewing results in the main analysis. An α of .05 was used to determine statistical significance. Bonferroni corrections were employed to compensate for family-wise error associated with 35 comparative odds ratios.

### Rate of Testing Positive After Initially Testing Negative

To assess the actual impact of adherence with serial testing, 2 rates (1 for patients with symptoms and 1 for patients without symptoms) were calculated to determine the percentage of COVID-19 cases that were not detected on the first Ag-RDT but were detected subsequently by serial testing.

### Serial Testing of Patients With Symptoms

To estimate the rate of episodes whereby patients with symptoms and initially negative Ag-RDTs subsequently tested positive during serial testing, testing episodes with a first Ag-RDT in a patient with symptoms that were followed by any second test were compiled. Then, the fraction of the second tests that were positive and negative was calculated, and the fraction of each group (positive and negative second tests) that was adherent to the FDA serial testing instructions was calculated.

### Serial Testing of Patients Without Symptoms

To estimate the rate of episodes whereby patients with initially negative Ag-RDTs subsequently tested positive during serial testing, testing episodes with patients without symptoms who had negative first and second Ag-RDTs, and a third test were compiled. Then, the fraction of the third tests that were positive was calculated, and the fraction of each group (positive and negative third tests) that was adherent to the FDA serial testing instructions was calculated.

### Software

We used R (version 4.4.1, R Foundation for Statistical Computing, Vienna, Austria) for all statistical analyses.

### Ethical Considerations

This study is not human subjects research because it only used data from public health surveillance activities [17]. Therefore, this study was exempt from requiring the Johns Hopkins University’s Institutional Review Board approval. This activity was reviewed by CDC, deemed not research, and was conducted consistent with applicable federal law and CDC policy [18].

## Results

### Patient Characteristics

Of the 61,356,012 ICATT testing records, a total of 2,929,477 unique records representing 2,189,464 patients with Ag-RDTs between August 12, 2022, and January 14, 2025, were included in the analysis of second-test adherence (Figure 1). The majority of records were excluded for falling outside the study window (n=13,296,938), followed by missing clinical data on self-reported symptom status (n=745,151), and only having NAAT/RT-PCR tests (n=4,605,219). Included records were then collapsed into 2,738,918 “testing episodes.” There were an additional 197 episodes removed since they were assumed to be screening testing because the total duration of the testing episode exceeded 30 days and the number of tests per day within the episode exceeded 0.2. In addition, testing episodes were excluded if the first test was a NAAT/RT-PCR test (n=150,595) or a positive antigen test (n=51,563). Finally, episodes were summarized by patient ID, with 347,099 episodes associated with another testing episode with the same patient ID, leaving 2,189,464 patients for analysis of second-test adherence. To summarize episodes by patient ID, rates were calculated for the fraction of testing episodes in which the patient was symptomatic and adherent for the second test or third test. In addition, the first testing record in the line-level dataset with a given patient ID was used as the source of all demographic information.

Of the patients included in the final analytic sample of second-test adherence (Table 1), the majority were adults (1,676,033/2,189,464, 76.5%), non-Hispanic or Latino (1,583,019/2,189,464, 72.3%), and tested at a CVS location (2,153,391/2,189,464, 98.4%; Table 1). Approximately 60% (1,312,464/2,189,464) of patients were women, and 14% (296,851/2,189,464) were Black or African American. Geographically, the majority of patients were from the South (1,251,495/2,189,464, 57.2%), followed by the Midwest (441,296/2,189,464, 20.2%) and the Northeast (297,278/2,189,464, 13.6%). Patients with private insurance comprised 70.5% (1,543,902/2,189,464) of the patient cohort. Uninsured patients represented 12.1% (264,071/2,189,464), Medicaid patients represented 7.9% (173,486/2,189,464), and Medicare patients represented 8% (176,015/2,189,464).

The analysis of third-test adherence followed the same process as second-test adherence for compiling testing episodes (Figure 1), but additional criteria were applied to exclude episodes inappropriate for analysis of third-test adherence. These exclusion criteria included having no follow-up second test (n=2,494,989), being symptomatic at the second antigen test (n=18,966), having a positive second antigen test (n=8077), and having a second NAAT/RT-PCR test (n=7112). Finally, episodes were summarized by patient ID, with 606 episodes associated with another testing episode with the same patient ID, leaving 6813 patients for analysis of third-test adherence.

Of the 6813 patients included in the final analytic sample of third-test adherence (Table 2), the majority were adults (5237/6813, 76.9%), non-Hispanic or Latino (4465/6813, 65.5%), and tested at a CVS location (6664/6813, 97.8%; Table 2). Approximately 60% (3939/6813) of participants were women, and 23% (1560/6813) were Black or African American. Geographically, the largest proportion of patients was from the South (3202/6813, 47%), followed by the Northeast (1570/6813, 23%) and the Midwest (1116/6813, 16.4%). Patients with private insurance comprised 56% (3812/6813) of the patient cohort, uninsured patients represented 18.6% (1265/6813), Medicaid patients represented 12.4% (842/6813), and Medicare patients represented 12.4% (846/6813).

**Table 1:** A summary of clinical and demographic data for patients included in the second-test analysis (N=2,189,464).

Characteristic		Value
Symptomatic rate (%), mean (SD)		87.3 (32.8)
**Age group (years), n (%)**		
	Adult (18-64)	1,676,033 (76.5)
	Child (<18)	281,575 (12.9)
	Older adult (>65)	231,856 (10.6)
**Race, n (%)**		
	American Indian or Alaska Native	8896 (0.4)
	Asian	139,251 (6.4)
	Black or African American	296,851 (13.6)
	Native Hawaiian or Other Pacific Islander	6837 (0.3)
	Not reported	397,562 (18.2)
	Other	117,439 (5.4)
	White	1,222,628 (55.8)
**Ethnicity, n (%)**		
	Hispanic/Latino	358,182 (16.4)
	Not Hispanic/Latino	1,583,019 (72.3)
	Not reported	248,263 (11.3)
**Region, n (%)**		
	Midwest	441,296 (20.2)
	Northeast	297,278 (13.6)
	Other/unspecified	917 (0.0)
	South	1,251,495 (57.2)
	West	198,478 (9.1)
**Sex, n (%)**		
	Female	1,312,464 (59.9)
	Male	869,172 (39.7)
	Undetermined	7828 (0.4)
**Insurance Status, n (%)**		
	Cash	29,013 (1.3)
	HRSA^a^	2961 (0.1)
	Medicaid	173,486 (7.9)
	Medicare	176,015 (8)
	Not Reported	16 (0.0)
	Private insurance	1,543,902 (70.5)
	Uninsured	264,071 (12.1)
**Site Contractor, n (%)**		
	CVS	2,153,391 (98.4)
	eTrueNorth	494 (0.0)
	Walgreens	35,579 (1.6)
**Year quarter, n (%)**		
	2022Q3	223,654 (10.2)
	2022Q4	363,206 (16.6)
	2023Q1	360,292 (16.5)
	2023Q2	195,041 (8.9)
	2023Q3	231,471 (10.6)
	2023Q4	246,923 (11.3)
	2024Q1	219,990 (10.0)
	2024Q2	97,829 (4.5)
	2024Q3	134,493 (6.1)
	2024Q4	99,484 (4.5)
	2025Q1	17,081 (0.8)

^a^HRSA: Health Resources and Services Administration.

**Table 2:** A summary of clinical and demographic data for patients included in the third-test analysis (N=6813).

Characteristic		Value
Symptomatic rate (%), mean (SD)		87.3 (32.8)
**Age group (years), n (%)**		
	Adult (18-64)	5237 (76.9)
	Child (<18)	452 (6.6)
	Older adult (>65)	1124 (16.5)
**Race, n (%)**		
	White	2689 (39.5)
	American Indian or Alaska Native	41 (0.6)
	Asian	477 (7)
	Black or African American	1560 (22.9)
	Native Hawaiian or Other Pacific Islander	41 (0.6)
	Not reported	1675 (24.6)
	Other	330 (4.8)
**Ethnicity, n (%)**		
	Not Hispanic/Latino	4465 (65.5)
	Hispanic/Latino	1480 (21.7)
	Not reported	868 (12.7)
**Region, n (%)**		
	Northeast	1570 (23)
	Midwest	1116 (16.4)
	Other/Unspecified	1 (0.0)
	South	3202 (47)
	West	924 (13.6)
**Sex, n (%)**		
	Male	2844 (41.7)
	Female	3939 (57.8)
	Undetermined	30 (0.4)
**Insurance Status, n (%)**		
	Private insurance	3812 (56)
	Cash	33 (0.5)
	HRSA^a^	15 (0.2)
	Medicaid	842 (12.4)
	Medicare	846 (12.4)
	Uninsured	1265 (18.6)
**Site Contractor, n (%)**		
	CVS	6664 (97.8)
	eTrueNorth	6 (0.1)
	Walgreens	143 (2.1)
**Year quarter, n (%)**		
	2022Q3	1906 (28)
	2022Q4	1738 (25.5)
	2023Q1	1694 (24.9)
	2023Q2	473 (6.9)
	2023Q3	419 (6.2)
	2023Q4	212 (3.1)
	2024Q1	149 (2.2)
	2024Q2	62 (0.9)
	2024Q3	126 (1.8)
	2024Q4	31 (0.5)
	2025Q1	3 (0.0)

^a^HRSA: Health Resources and Services Administration.

### Patient and Testing Episode Adherence

At the patient level, second-test adherence was rare. Less than 1% (20,290/2,189,464) of patients included in the second-test adherence analysis were adherent to the instructions for a second test after an initial negative Ag-RDT, and 98.2% (2,149,292/2,189,464) had no second test documented in ICATT and therefore were classified as nonadherent in this analysis. At the testing-episode level, of the 2,536,563 testing episodes eligible for second-test adherence analysis, over 98% (n=2,494,989) were not adherent for either the second or third test (Table 3). The reason for lack of adherence was almost entirely a lack of documented follow-on testing in ICATT (2,494,989/2,536,563, 98.4% for the second test and 5894/7419, 79.4% for the third test), which may reflect loss to follow-up or follow-up testing done outside the program in addition to true nonadherence. Among testing episodes with a second test and only a negative Ag-RDT (n=41,574), the average time between tests was 4.15 (SD 2.59) days. Roughly half (20,481/41,574, 49.3%) of those testing episodes were adherent, either with a second Ag-RDT performed 2-3 days after the first negative Ag-RDT (13,369/41,574, 32.2%) or with a NAAT/RT-PCR test (7112/41,574, 17.1%). However, of the remaining 50.7% of included tests, 40.2% (16,700/41,574) received the follow-up Ag-RDT after the required window, while 10.6% (4393/41,574) received the follow-up Ag-RDT too soon. Patients who received a second Ag-RDT waited an average of 4.15 (SD 2.59) days after their first Ag-RDT.

Among the 7419 testing episodes with a recommended third test per FDA instructions, a total of 20.6% (1525/7419) had a third test. Of those, 75.9% (1157/1525) were nonadherent due to late Ag-RDT testing, 4.5% (69/1525) were nonadherent due to early antigen testing, 14% (214/1525) were adherent from on-time antigen testing, and 5.6% (85/1525) were adherent from NAAT/RT-PCR testing at the third testing encounter. Patients who received a third Ag-RDT waited an average of 5.84 (SD 2.25) days after their second Ag-RDT.

Similar results (Table 4) were found for serial testing adherence of testing episodes used for the expanded model that restricted the inclusion criteria to testing records with complete reporting for the following clinical variables: symptom status, recent COVID-19 contact, having had COVID-19 within 90 days, and having received a vaccine.

**Table 3:** A summary of testing episode adherence for the second and third test analyses. Episodes with no documented follow-up test in Increasing Community Access to Testing, Treatment, and Response (ICATT) were classified as nonadherent for analytic purposes; this category may include loss to follow-up or follow-up testing outside ICATT.

Characteristic		Value
**Second-test adherence, n/N (%)**		
	Adherent: second test antigen on-time	13,369/2,536,563 (0.5)
	Adherent: second test PCR^a^	7112/2,536,563 (0.3)
	Nonadherent: no follow-up second test	2,494,989/2,536,563 (98.4)
	Nonadherent: second test antigen early	4393/2,536,563 (0.2)
	Nonadherent: second test antigen late	16,700/2,536,563 (0.7)
**Third-test adherence, n/N (%)**		
	Adherent: third test antigen on-time	214/7419 (2.9)
	Adherent: third test PCR	85/7419 (1.1)
	Nonadherent: no follow-up third test	5894/7419 (79.4)
	Nonadherent: third test antigen early	69/7419 (0.9)
	Nonadherent: third test antigen late	1157/7419 (15.6)
**Timing between tests, mean (SD)**		
	Days between test 1 and 2	4.15 (2.59)
	Days between test 2 and 3	5.84 (2.25)

^a^PCR: polymerase chain reaction.

**Table 4:** Serial testing adherence of testing episodes for the expanded model, which required clinical variables in addition to symptom status to be reported. Episodes with no documented follow-up test in Increasing Community Access to Testing, Treatment, and Response (ICATT) were classified as nonadherent for analytic purposes; this category may include loss to follow-up or follow-up testing outside ICATT.

Characteristic		Value
**Second-test adherence, n/N (%)**		
	Adherent: second antigen test, on time	572/140,461 (0.4)
	Adherent: second PCR^a^ test	547/140,461 (0.4)
	Nonadherent: no follow-up second test	138,330/140,461 (98.5)
	Nonadherent: second antigen test, early	252/140,461 (0.2)
	Nonadherent: second antigen test, late	760/140,461 (0.5)
**Third-test adherence, n/N (%)**		
	Adherent: third antigen test, on time	8/450 (1.8)
	Adherent: third PCR test	3/450 (0.7)
	Nonadherent: no follow-up third test	343/450 (76.2)
	Nonadherent: third antigen test, early	6/450 (1.3)
	Nonadherent: third antigen test, late	90/450 (20)
**Time between tests, mean (SD)**		
	Days between tests 1 and 2	3.85 (2.26)
	Days between tests 2 and 3	3.53 (2.26)

^a^PCR: polymerase chain reaction.

### Second-Test Adherence: Demographic Variables

Multiple clinical and patient demographics were significantly associated with second-test adherence (Table 5). Patients with symptoms had 47% lower odds (adjusted odds ratio [AOR] 0.53, 95% CI 0.51-0.55; *P*<.001) of adherence compared with those who were without symptoms. Both children (AOR 0.48, 95% CI 0.45-0.51; *P*<.001) and adults aged 65 years and older (AOR 0.83, 95% CI 0.77-0.88; *P*<.001) had lower odds of adherence compared with adults. In contrast, both Asian (AOR 1.28, 95% CI 1.21-1.36; *P*<.001) and Black (AOR 1.17, 95% CI 1.12-1.23; *P*<.001) individuals had higher odds of adherence compared with White individuals. Hispanic or Latino individuals had 9% higher odds of adherence (AOR 1.09, 95% CI 1.03-1.16; *P*=.003) compared with non-Hispanic individuals; however, this association did not remain statistically significant after correction for multiple comparisons. All geographic regions had lower odds of adherence than the Northeast, with the largest difference observed in the South (AOR 0.82, 95% CI 0.79-0.86; *P*<.001). Women had 8% higher odds of adherence (AOR 1.08, 95% CI 1.05-1.11; *P*<.001) compared with men. Both patients with Medicare (AOR 1.11, 95% CI 1.03-1.20; *P*=.005) and patients with Medicaid (AOR 1.14, 95% CI 1.07-1.20; *P*<.001) had higher odds of adherence than those with private insurance; however, the association for Medicare did not remain statistically significant after correction for multiple comparisons. Estimates for the site contractor eTrueNorth (Table 5) are based on small sample sizes (Table 1), as reflected by wider CIs. Adherence significantly attenuated over time since guideline implementation, ranging from 17% lower odds in the quarter immediately following guideline implementation (AOR 0.83, 95% CI 0.79-0.87; *P*<.001) to 92% lower odds of adherence more than 2 years later (AOR 0.08, 95% CI 0.05-0.13; *P*<.001).

**Table 5:** Fractional logit regression model of second-test adherence.

Characteristic			OR^a^ (95% CI)		*P* value
Symptomatic rate			0.53 (0.51-0.55)		<.001
**Age group (years; reference: adult, 18-64)**					
	Child (<18)	0.48 (0.45-0.51)		<.001	
	Older adult (>65)	0.83 (0.77-0.88)		<.001	
**Race (reference: White)**					
	American Indian or Alaska Native	1.07 (0.84-1.33)		.57	
	Asian	1.28 (1.21-1.36)		<.001	
	Black or African American	1.17 (1.12-1.23)		<.001	
	Native Hawaiian or Other Pacific Islander	1.21 (0.94-1.53)		.13	
	Not reported	1.16 (1.10-1.24)		<.001	
	Other	1.00 (0.92-1.08)		.93	
**Ethnicity (reference: not Hispanic/Latino)**					
	Hispanic/Latino	1.09 (1.03-1.16)		.003	
	Not reported	0.83 (0.78-0.88)		<.001	
**Region (reference: Northeast)**					
	Midwest	0.83 (0.79-0.88)		< .001	
	Other/unspecified	0.18 (0.03-0.55)		.01	
	South	0.82 (0.79-0.86)		<.001	
	West	0.93 (0.88-0.99)		.02	
**Sex (reference: male)**					
	Female	1.08 (1.05-1.11)		<.001	
	Undetermined	1.25 (0.99-1.55)		.049	
**Insurance Status (reference: private insurance)**					
	Cash	1.16 (0.98-1.37)		.08	
	HRSA^b^	1.23 (0.86-1.70)		.24	
	Medicaid	1.14 (1.07-1.20)		<.001	
	Medicare	1.11 (1.03-1.20)		.005	
	Not Reported	1.67 (0.09-9.33)		.63	
	Uninsured	1.01 (0.96-1.05)		.74	
**Site Contractor (reference: CVS)**					
	eTrueNorth	4.97 (2.90-7.91)		<.001	
	Walgreens	0.87 (0.76-1.00)		.06	
**Year quarter (reference: 2022Q3)**					
	2022Q4	0.83 (0.79-0.87)		<.001	
	2023Q1	0.78 (0.75-0.82)		<.001	
	2023Q2	0.51 (0.48-0.54)		<.001	
	2023Q3	0.51 (0.48-0.54)		<.001	
	2023Q4	0.34 (0.31-0.36)		<.001	
	2024Q1	0.27 (0.25-0.29)		<.001	
	2024Q2	0.23 (0.20-0.26)		<.001	
	2024Q3	0.28 (0.25-0.31)		<.001	
	2024Q4	0.15 (0.12-0.17)		<.001	
	2025Q1	0.08 (0.05-0.13)		<.001	

^a^OR: odds ratio.

^b^HRSA: Health Resources and Services Administration.

### Second-Test Adherence Expanded Model: Demographic and Clinical Variables

**Figure 3:**
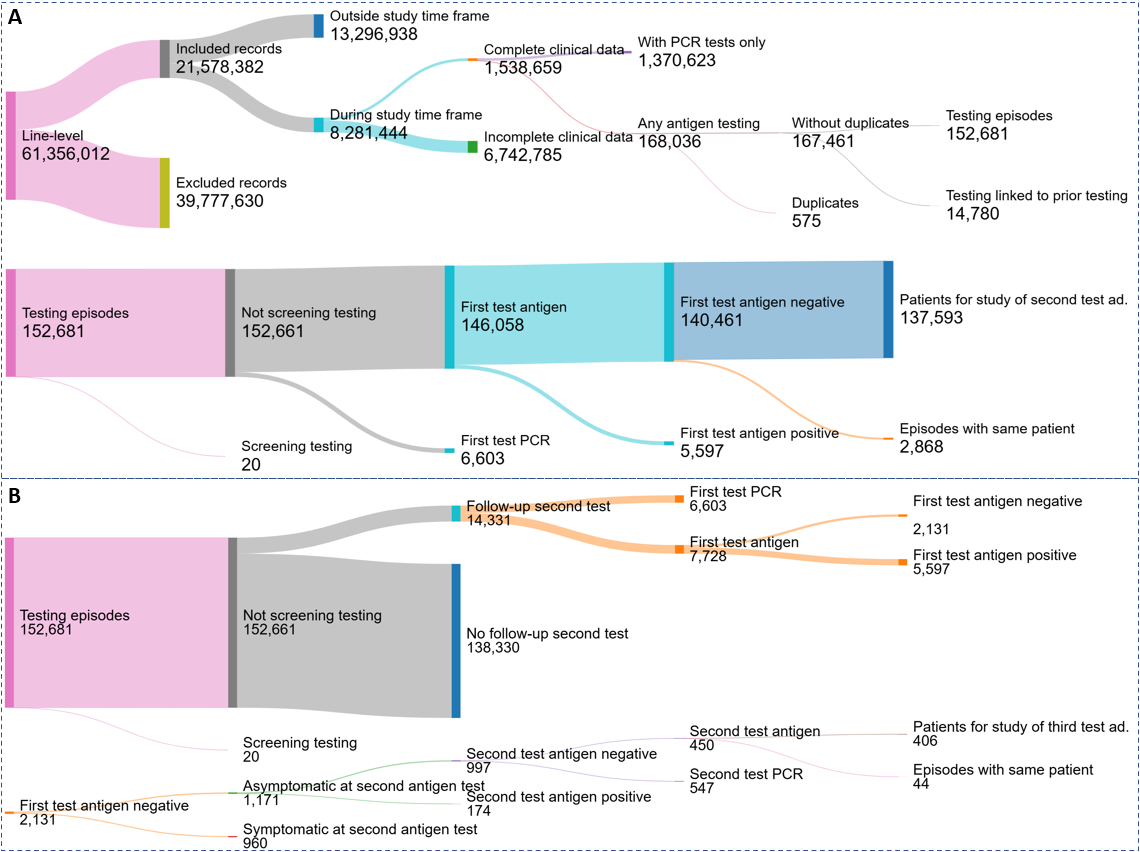
Patient flow diagram for the second-test and third-test adherence analyses (expanded model). (A) Flow for the second-test adherence analysis; (B) Flow for the third-test adherence analysis. The term polymerase chain reaction (PCR) is used in the figure to denote any nucleic acid amplification test (NAAT).

There were 137,593 patients (Figure 3) included in the expanded model with complete demographic and clinical information (Table 6), representing a substantially smaller complete-case subset of the primary analytic sample. After reducing the model to this sample and adding in the 3 clinical variables (recent COVID-19 contact, COVID-19 within 90 days, and vaccination status), many of the demographic variables (sex, insurance status, and symptom status) were no longer statistically significant (Table 7). However, the newly added clinical variables were significantly associated with the outcome. For example, testing episodes among patients with a recent COVID-19 contact had 87% higher odds (AOR 1.87, 95% CI 1.64-2.13; *P*<.001) of adherence compared with patients without recent COVID-19 contact. In contrast, testing episodes among patients who had COVID-19 within the past 90 days had 34% lower odds (AOR 0.66, 95% CI 0.56-0.76; *P*<.001) of adherence. Vaccination status (AOR 1.07, 95% CI 0.92-1.26; *P*=.40) was not significantly associated with adherence.

Among demographic variables, children had 46% lower odds of adherence than adults (AOR 0.54, 95% CI 0.40-0.72; *P*<.001), while older adult patients were no longer statistically different from adults (AOR 0.89, 95% CI 0.68-1.16; *P*=.40). Asian individuals had 33% higher odds of adherence (AOR 1.33, 95% CI 1.05-1.67; *P*=.02) compared with White individuals; however, this association did not remain statistically significant after correction for multiple comparisons, and all other racial categories were not statistically significant. Testing episodes in the quarter immediately following the issuance of the FDA recommendations showed the opposite direction of association, with 20% higher odds of adherence (AOR 1.20, 95% CI 1.04-1.38; *P*=.01) compared with the quarter of policy implementation; however, this association did not remain statistically significant after correction for multiple comparisons.

**Table 6:** Patient demographic and clinical characteristics for the expanded model of second-test adherence study (N=137,593).

Characteristic		Value
**Clinical characteristics (%), mean (SD)**		
	Symptomatic rate	82.0 (38.3)
	Recent COVID-19 contact rate	50.0 (49.8)
	Had COVID-19 within 90 days rate	55.0 (49.6)
	Has received vaccine rate	77.0 (42.3)
**Age group (years), n (%)**		
	Adult (18-64)	112,601 (81.8)
	Child (<18)	10,204 (7.4)
	Older Adult (>65)	14,788 (10.7)
**Race, n (%)**		
	White	69,085 (50.2)
	American Indian or Alaska Native	798 (0.6)
	Asian	9631 (7.0)
	Black or African American	26,188 (19.0)
	Native Hawaiian or Other Pacific Islander	502 (0.4)
	Not reported	26,343 (19.1)
	Other	5046 (3.7)
**Ethnicity, n (%)**		
	Not Hispanic/Latino	97,082 (70.6)
	Hispanic/Latino	27,312 (19.8)
	Not reported	13,199 (9.6)
**Region, n (%)**		
	Northeast	19,386 (14.1)
	Midwest	26,840 (19.5)
	Other/unspecified	146 (0.1)
	South	74,331 (54.0)
	West	16,890 (12.3)
**Sex, n (%)**		
	Male	55,696 (40.5)
	Female	81,242 (59.0)
	Undetermined	655 (0.5)
**Insurance status, n (%)**		
	Private insurance	82,835 (60.2)
	Cash	2667 (1.9)
	HRSA^a^	220 (0.2)
	Medicaid	11,377 (8.3)
	Medicare	10,071 (7.3)
	Uninsured	30,423 (22.1)
**Site Contractor, n (%)**		
	CVS	119,592 (86.9)
	Walgreens	18,001 (13.1)
**Year quarter, n (%)**		
	2022Q3	46,086 (33.5)
	2022Q4	34,580 (25.1)
	2023Q1	20,164 (14.7)
	2023Q2	4614 (3.4)
	2023Q3	7776 (5.7)
	2023Q4	5027 (3.7)
	2024Q1	3930 (2.9)
	2024Q2	944 (0.7)
	2024Q3	8224 (6.0)
	2024Q4	5326 (3.9)
	2025Q1	922 (0.7)

^a^HRSA: Health Resources and Services Administration.

**Table 7:** Fractional logit regression for the expanded model of second-test adherence.

Characteristic			OR^a^ (95% CI)		*P* value
**Clinical characteristics**					
	Symptomatic rate	0.91 (0.78-1.05)		.20	
	Recent COVID-19 contact rate	1.87 (1.64-2.13)		<.001	
	Had COVID-19 within 90 days rate	0.66 (0.56-0.76)		<.001	
	Has received vaccine rate	1.07 (0.92-1.26)		.40	
**Age group (years; reference: adult, 18-64)**					
	Child (<18)	0.54 (0.40-0.72)		<.001	
	Older Adult (>65)	0.89 (0.68-1.16)		.40	
**Race (reference: White)**					
	American Indian or Alaska Native	0.53 (0.13-1.38)		.27	
	Asian	1.33 (1.05-1.67)		.02	
	Black or African American	1.03 (0.86-1.22)		.77	
	Native Hawaiian or Other Pacific Islander	0.57 (0.09-1.79)		.43	
	Not reported	0.85 (0.67-1.09)		.21	
	Other	1.07 (0.76-1.47)		.69	
**Ethnicity (reference: not Hispanic/Latino)**					
	Hispanic/Latino	1.16 (0.92-1.44)		.20	
	Not reported	0.78 (0.59-1.02)		.08	
**Region (reference: Northeast)**					
	Midwest	0.71 (0.58-0.86)		<.001	
	Other/unspecified	1.00 (0.10-4.44)		.88	
	South	0.70 (0.60-0.83)		<.001	
	West	0.57 (0.44-0.73)		<.001	
**Sex (reference: male)**					
	Female	1.02 (0.90-1.16)		.72	
	Undetermined	1.06 (0.35-2.48)		.90	
**Insurance Status (reference: private insurance)**					
	Cash	0.00 (0.00-0.00)		.94	
	HRSA^b^	2.78 (1.09-5.77)		.02	
	Medicaid	1.07 (0.86-1.32)		.56	
	Medicare	0.92 (0.67-1.25)		.60	
	Uninsured	0.89 (0.74-1.06)		.18	
**Site contractor (reference: CVS)**					
	Walgreens	0.96 (0.72-1.25)		.74	
**Year Quarter (reference: 2022Q3)**					
	2022Q4	1.20 (1.04-1.38)		.01	
	2023Q1	1.05 (0.86-1.29)		.62	
	2023Q2	0.63 (0.39-0.96)		.04	
	2023Q3	0.11 (0.04-0.22)		<.001	
	2023Q4	0.03 (0.00-0.14)		<.001	
	2024Q1	0.08 (0.01-0.26)		<.001	
	2024Q2	0.20 (0.01-0.89)		.11	
	2024Q3	0.15 (0.07-0.29)		<.001	
	2024Q4	0.38 (0.21-0.67)		.001	
	2025Q1	0.00 (0.00-0.00)		.97	

^a^OR: odds ratio.

^b^HRSA: Health Resources and Services Administration.

### Third-Test Adherence: Demographic Variables

Some similar trends among demographic variables were observed for third-test adherence (Table 8). Patients with symptoms had 35% lower odds of adherence compared with those without symptoms (AOR 0.65, 95% CI 0.45-0.91; *P*=.02), and children had 50% lower odds of adherence than adults (AOR 0.50, 95% CI 0.24-0.94; *P*=.05); however, these associations did not remain statistically significant after correction for multiple comparisons. Asian individuals had 53% lower odds of adherence than White individuals (AOR 0.47, 95% CI 0.24-0.82; *P*=.01); however, this association did not remain statistically significant after correction for multiple comparisons, and all other racial categories were not statistically significant. Among geographic regions, the only significant difference was observed in the West, which had 47% higher odds of adherence than the Northeast (AOR 1.47, 95% CI 1.00-2.15; *P*=.048); however, this association did not remain statistically significant after correction for multiple comparisons. Ethnicity, sex, insurance status, site contractor, and temporality were not statistically significant. Similar to second-test adherence, the odds of third-test adherence also declined over time.

**Table 8:** Fractional logit regression model of third-test adherence.

Characteristic		OR^a^ (95% CI)	*P* value
Symptomatic rate		0.65 (0.45-0.91)	.02
**Age group (years; reference: adult, 18-64)**			
	Child (<18)	0.50 (0.24-0.94)	.05
	Older adult (>65)	0.94 (0.60-1.44)	.79
**Race (reference: White)**			
	American Indian or Alaska Native	0.00 (0.00-247,000)	.98
	Asian	0.47 (0.24-0.82)	.01
	Black or African American	0.75 (0.52-1.05)	.20
	Native Hawaiian or Other Pacific Islander	0.00 (0.00-373,000)	.98
	Not reported	0.74 (0.47-1.16)	.18
	Other	1.22 (0.71-2.00)	.46
**Ethnicity (reference: not Hispanic/Latino)**			
	Hispanic/Latino	0.99 (0.62-1.53)	.96
	Not reported	1.59 (1.03-2.41)	.03
**Region (reference: Northeast)**			
	Midwest	1.06 (0.72-1.54)	.78
	Other/unspecified	7.09E-07 (N/A^b^-infinity)	>.99
	South	0.79 (0.57-1.10)	.16
	West	1.47 (1.00-2.15)	.048
**Sex (reference: male)**			
	Female	1.09 (0.85-1.40)	.50
	Undetermined	0.00 (0.00-1,410,000,000)	.98
**Insurance status (reference: private insurance)**			
	Cash	0.00 (0.00-95,800,000)	.98
	HRSA^c^	5.60E-07 (N/A-infinity)	.99
	Medicaid	1.17 (0.79-1.70)	.41
	Medicare	0.78 (0.46-1.31)	.36
	Uninsured	1.17 (0.84-1.62)	.34
**Site contractor (reference: CVS)**			
	eTrueNorth	1.41E-06 (N/A-infinity)	.99
	Walgreens	0.66 (0.22-1.53)	.38
**Year Quarter (ref: 2022Q3)**			
	2022Q4	0.87 (0.63-1.19)	.38
	2023Q1	0.77 (0.55-1.07)	.13
	2023Q2	0.95 (0.57-1.52)	.84
	2023Q3	0.75 (0.40-1.30)	.33
	2023Q4	0.64 (0.24-1.36)	.29
	2024Q1	0.31 (0.05-1.01)	.11
	2024Q2	0.00 (0.00-106)	.98
	2024Q3	0.39 (0.06-1.27)	.20
	2024Q4	0.85 (0.05-4.15)	.88
	2025Q1	5.87E-07 (N/A-infinity)	>.99

^a^OR: odds ratio.

^b^N/A: not applicable.

^c^HRSA: Health Resources and Services Administration.

### Third-Test Adherence Expanded Model: Demographic and Clinical Variables

There were 406 patients included in the expanded model with complete demographic and clinical variables (Table 9), reflecting a markedly reduced complete-case sample. After adding additional clinical variables into the model and restricting the analysis to this sample, the majority of demographic variables were no longer statistically significant (Table 10). The only significant relationship was observed for recent COVID-19 contacts, as those individuals had 1643% higher odds of adherence (AOR 17.43, 95% CI 2.14-546.41; *P*=.03); however, the wide CI around this estimate indicates limited precision, and the association did not remain statistically significant after correction for multiple comparisons.

**Table 9:** Patient demographic and clinical characteristics for the expanded model of the third-test adherence study (N=406).

Characteristic		Value
**Clinical characteristics (%), mean (SD)**		
	Symptomatic rate	22.0 (41.4)
	Recent COVID-19 contact rate	57.0 (49.2)
	Had COVID-19 within 90 days rate	48.0 (49.5)
	Has received vaccine rate	62.0 (48.5)
**Age group (years), n (%)**		
	Adult (18-64)	327 (80.5)
	Child (<18)	12 (3)
	Older adult (>65)	67 (16.5)
**Race, n (%)**		
	White	196 (48.3)
	American Indian or Alaska Native	0 (0)
	Asian	25 (6.2)
	Black or African American	99 (24.4)
	Native Hawaiian or Other Pacific Islander	2 (0.5)
	Not reported	71 (17.5)
	Other	13 (3.2)
**Ethnicity, n (%)**		
	Not Hispanic/Latino	314 (77.3)
	Hispanic/Latino	61 (15)
	Not reported	31 (7.6)
**Region, n (%)**		
	Northeast	122 (30)
	Midwest	69 (17)
	South	176 (43.3)
	West	39 (9.6)
**Sex, n (%)**		
	Male	145 (35.7)
	Female	257 (63.3)
	Undetermined	4 (1)
**Insurance status, n (%)**		
	Private insurance	257 (63.3)
	Cash	1 (0.2)
	HRSA^a^	0 (0)
	Medicaid	54 (13.3)
	Medicare	40 (9.9)
	Uninsured	54 (13.3)
**Site contractor, n (%)**		
	CVS	365 (89.9)
	Walgreens	41 (10.1)
**Year Quarter, n (%)**		
	2022Q3	209 (51.5)
	2022Q4	123 (30.3)
	2023Q1	57 (14.0)
	2023Q2	6 (1.5)
	2023Q3	5 (1.2)
	2023Q4	2 (0.5)
	2024Q1	1 (0.2)
	2024Q2	1 (0.2)
	2024Q3	1 (0.2)
	2024Q4	1 (0.2)
	2025Q1	0 (0)

^a^HRSA: Health Resources and Services Administration.

**Table 10:** Fractional logit regression for the expanded model of third-test adherence. Note: some estimates are unstable due to sparse cells in the expanded third-test model.

Characteristic		OR^a^ (95% CI)	*P* value
**Clinical characteristics**			
	Symptomatic rate	2.40 (0.39-13.56)	.32
	Recent COVID-19 contact rate	17.43 (2.14-546.41)	.03
	Had COVID-19 within 90 days rate	0.85 (0.14-4.80)	.85
	Has received vaccine rate	0.30 (0.05-1.59)	.16
**Age group (years; reference: adult, 18-64)**			
	Child (<18)	0 (N/A^b^-infinity)	>.99
	Older Adult (65+)	2.00 (0.08-20.61)	.59
**Race (reference: White)**			
	Asian	2.49 (0.09-35.39)	.52
	Black or African American	0.82 (0.10-4.73)	.83
	Native Hawaiian or Other Pacific Islander	0 (N/A-infinity)	>.99
	Not reported	0.09 (0.00-2.15)	.16
	Other	9.62E-09 (0-infinity)	>.99
**Ethnicity (reference: not Hispanic/Latino)**			
	Hispanic/Latino	1.80 (0.06-33.80)	.70
	Not reported	20.61 (0.65-714.67)	.08
**Region (reference: Northeast)**			
	Midwest	0 (N/A-infinity)	>.99
	South	0.68 (0.12-3.94)	.66
	West	1.20 (0.09-13.29)	.88
**Sex (reference: male)**			
	Female	0.99 (0.19-6.11)	.99
	Undetermined	0.38 (0-infinity)	>.99
**Insurance status (reference: private insurance)**			
	Cash	0 (N/A-infinity)	>.99
	Medicaid	0.32 (0.01-3.14)	.39
	Medicare	0 (N/A-infinity)	>.99
	Uninsured	0 (0-infinity)	>.99
**Site contractor (reference: CVS)**			
	Walgreens	0.92 (0.08-7.14)	.94
**Year Quarter (ref: 2022Q3)**			
	2022Q4	0.21 (0.01-1.46)	.18
	2023Q1	0.38 (0.01-4.15)	.47
	2023Q2	6.14 (0.20-114.87)	.23
	2023Q3	0 (0-infinity)	>.99
	2023Q4	2.34 (N/A-infinity)	>.99
	2024Q1	0.64 (0-infinity)	>.99
	2024Q2	7.98 (N/A-infinity)	>.99
	2024Q3	N/A (N/A-N/A)	N/A
	2024Q4	0 (N/A-infinity)	>.99

^a^OR: odds ratio.

^b^N/A: not applicable.

### Analysis of the Rate of Testing Positive After Initially Testing Negative

#### Serial Testing of Patients With Symptoms

Among 2,025,975 episodes with a negative first Ag-RDT, 1,742,160 (86%) were symptomatic at the first test. Of the episodes with patients with symptoms, a total of 29,315 (1.7%) had a second test, and 7310 (24.9%) of those receiving a second Ag-RDT were positive. Among this group, a total of 4195 (57.4%) of the episodes were adherent with serial testing (eg, an Ag-RDT taken 2-3 days after the first test or an RT-PCR test). Of the 22,005 (75.1%) episodes with a negative second Ag-RDT, a total of 10,818 (49.2%) were adherent with serial testing. Therefore, 24.9% (7310/29,315) of episodes among patients with symptoms tested negative on the first antigen test (95% CI 24.4%-25.4%) and positive on the second test when performing serial testing with 2 antigen tests.

#### Serial Testing of Patients Without Symptoms

Among 2,025,975 episodes with a negative first Ag-RDT, a total of 7792 (0.39%) were asymptomatic at the first and second tests, and 5957 (0.29%) were also negative at the second Ag-RDT. Among the 5957 episodes among patients without symptoms and with 2 negative Ag-RDTs, a total of 1435 (24.1%) had a third test, of whom 60 (4.2%) were positive, and 1375 (95.8%) were negative for the third Ag-RDT. Of the 60 episodes with a positive third test, 36 (60%) were adherent with serial testing. Of the 1375 episodes with a negative third test, 246 (17.9%) were adherent with serial testing. Therefore, the rate of testing positive through serial testing for this population was 4.2% (60/1435, 95% CI 3.2%-5.2%). In addition, serial testing was also effective for 24 patients without symptoms who were positive on the third test but nonadherent, indicating that serial testing can still be effective in situations where patients do not strictly adhere to testing timelines.

## Discussion

### Overview

This study is the first to evaluate serial Ag-RDT testing adherence following the FDA’s safety communication issued in August 2022. Among 2,189,464 patients who received at least 1 Ag-RDT through the CDC ICATT program, less than 2% also received a follow-up test captured by the program. Accordingly, the large group classified as nonadherent because no follow-up test was observed likely includes a mixture of true nonadherence, loss to follow-up, and follow-up testing done outside ICATT. Multiple demographic variables (including age, race, and region) were significantly associated with adherence, although some variables (eg, insurance, sex, or timeliness relative to the issuance of the recommendations) were only significant for second-test adherence. In the expanded model that included 3 clinical variables, however, the majority of demographic variables were no longer statistically significant. Instead, interaction with a recent COVID-19 contact significantly increased the odds of second- and third-test adherence, while patients who had COVID-19 themselves within the past 90 days were less likely to be adherent. Taken together, these results suggest that clinical variables and temporal nearness to policy issuance and likelihood of exposure (due to a substantial reduction in cases since policy issuance [19,20]) are more closely related to adherence than nonclinical demographic variables.

Within this study, there were only a few demographic differences associated with serial testing adherence, and these distinctions differed from those identified in other studies. In university environments, women were more likely than men to adhere to serial testing policies [11,14], while Asian and Black individuals were less likely to adhere [11]. This partially aligned with the results presented here; while women were also found to be more adherent than men for their second test, so were Asian and Black individuals (although both became statistically insignificant after adjusting for additional clinical variables). These differences may be attributable to the setting of the study (eg, a university setting in a relatively closed environment vs a broader geographic sample) or the time frame in which the studies occurred and the corresponding availability of resources. In particular, over-the-counter Ag-RDT tests were not approved by the FDA until December 2020, which was after the Wisconsin university study occurred [11,21]. As a result, patients in that study may have been more likely to adhere to serial testing because they did not have accessible over-the-counter tests.

In contrast, patients in this study may appear to be nonadherent with serial testing when, in actuality, they took a second Ag-RDT that was not captured by the ICATT program. As a result, the availability of at-home Ag-RDTs is a notable confounding variable. However, it is unlikely that all patients who did not receive a follow-up Ag-RDT within the ICATT data took an at-home Ag-RDT. A previous cross-sectional online survey found that approximately 20% of participants had engaged in at-home COVID-19 testing by March 2022, especially those who had either a COVID-19 exposure or COVID-19-like symptoms [22]. Even if a similar proportion of testing episodes without a second Ag-RDT in the ICATT data had an Ag-RDT, that would still leave over 1,995,991 testing episodes without a follow-up test. This may still be an underestimation because a significant portion of test takers were uninsured, and at-home testing rates are lower among these individuals [16,22].

In the expanded model that included only persons with complete clinical data, the majority of the demographic variables were no longer statistically significant (especially within the second-test adherence model). This suggests that a patient’s recent COVID-19 environment is more of a driver to testing than various demographic variables. Furthermore, the conflicting directionality of recent COVID-19 contact (which was associated with higher adherence) and recent COVID-19 status (which was associated with lower adherence) could be attributable to the rationale behind why an individual sought ICATT testing. For example, a patient who recently had COVID-19 may seek out an ICATT test to confirm that they no longer have the disease, as opposed to testing for a new instance of the disease. In contrast, patients with a recent COVID-19 contact may be more motivated to test because of concern that they may actively have COVID-19. These expanded-model findings should be interpreted cautiously because they were based on complete-case analyses using substantially smaller samples than the primary models. If the completeness of the clinical variables was nonrandom, the expanded-model estimates may be subject to selection bias. The smaller analytic samples, particularly for third-test adherence, also reduced precision and statistical power and may partly explain why some associations were attenuated or no longer statistically significant.

Interestingly, symptom status and vaccination status were not significant after adjusting for COVID-19 contact status, suggesting that actual contact with another patient may be more motivating than a person’s own symptoms or vaccination history. This aligns with findings from a scoping review that found that a person’s perception of having COVID-19 and motivation for testing are heavily influenced by their social environment [23]. This may also explain why adherence declined over time relative to the announcement of a new safety communication, as motivation to comply may be higher directly after the initial announcement occurs. In addition, independent pharmacies (eTrueNorth sites) showed significantly higher odds of test adherence than chain pharmacies. Similar results have been found in terms of medication adherence by independent pharmacy users [24]. Because all ICATT vendors were required to follow FDA regulations and manufacturers’ instructions for use, this difference is unlikely due to procedural variations and warrants further investigation. Overall, these results suggest the need for public health officials to make frequent reminders about changes in instructions to stress their importance and reinforce positive behaviors, although additional work is needed to understand the most effective messaging strategy and how over-the-counter testing may have played a role in serial testing adherence. Although we did not directly measure exposure to public health messaging or pharmacist (testing-site) counseling, the low adherence observed here suggests a need for future work on how serial-testing guidance is communicated to patients and how access to convenient follow-up testing can be improved during future pandemics. Although several ORs were statistically significant, many were close to 1 and therefore likely represent modest practical differences at the individual level. The more important public health finding is the uniformly low uptake of serial testing across the cohort, suggesting broad barriers to adherence rather than large disparities concentrated in a few subgroups. Taken together, these findings suggest that low adherence likely reflects a combination of behavioral factors, including perceived exposure risk and declining motivation over time, and structural factors, including the availability of at-home testing, differences in access, and possible variation in counseling across testing sites.

The analysis on the rate of testing positive through serial testing indicated that serial testing can reduce the risk of false-negative Ag-RDT results, aligning with the FDA safety communication. For patients with symptoms, serial testing (eg, a follow-up Ag-RDT or NAAT/RT-PCR test after the initial test) identified 24.9% of initially negative cases as positive upon retesting, underscoring the importance of multiple tests to catch infections missed by earlier tests. Adherence with serial testing was higher among those who tested positive on the second test (57.4%) compared with those who remained negative (49.2%). Of the 60 episodes with a positive third test, 36 (60%) were adherent with serial testing. Of the 1375 episodes with a negative third test, 246 (17.9%) were adherent with serial testing. For patients without symptoms, the rate of testing positive via serial testing was lower at 4.2%. This lower rate of testing positive for patients without symptoms is logical, as patients without symptoms are less likely to have COVID-19.

### Limitations

This study’s strength is its large sample size of over 2 million patients over a multiyear time frame. However, there are several limitations that are important to note. Testing data are limited to tests that occurred at vendor sites and do not include at-home tests or tests performed outside of ICATT, creating the potential for misclassification bias in the adherence outcome. As a result, some patients classified as nonadherent may in fact have completed follow-up testing outside the observed pharmacy network. Similarly, it is assumed that the first ICATT test is the start of an individual’s testing episode, when a patient may be using ICATT as a follow-up for a test they took at home or at a non-ICATT facility. In addition, observation of repeat testing was limited within the pharmacy chain because patient IDs were generated separately by vendors and were not used across vendors, so linking testing records for the same patient between different pharmacy chains was not possible. Finally, vendors performed testing according to the authorized instructions for use and counseled patients on the serial testing protocol; however, there was no measure of vendor compliance or patient compliance, and there was no measure of whether patients knew the importance of serial testing. Future work could seek to validate the findings presented here by measuring at-home testing rates or examining testing in other health care settings among demographic groups targeted by the ICATT program, as well as measuring knowledge of the serial testing safety communication.

Furthermore, ICATT is a nonprobability sample that targets underresourced communities; as a result, the findings presented here may not be representative of the broader US population. In addition, the study’s cut-off date extends well beyond the issued safety communication date, and it is possible that the likelihood of adherence due to the safety communication was lower for dates further out from the announcement. Third, a gender variable closely linked to sex was used to determine a patient’s sex because sex was not collected by the ICATT vendors. Finally, there was a high degree of missingness among self-reported clinical variables. Consequently, the expanded models were restricted to complete cases, and if the completeness of the clinical variables was nonrandom, those estimates may be affected by selection bias. The markedly smaller complete-case samples—especially for the third-test expanded model—also reduced precision and statistical power.

### Conclusion

This study is the first to evaluate adherence with SARS-CoV-2 serial Ag-RDT testing following the issuance of an FDA safety communication. Our results highlight the value of public-private programs such as ICATT, which not only directly delivered testing services to uninsured individuals during the COVID-19 pandemic but also provided a detailed source of data to evaluate the effectiveness of government safety communications, such as serial testing instructions for Ag-RDTs, as well as the real-world performance of medical countermeasures such as diagnostic testing. The CDC ICATT program can serve as a model for public health programs and data infrastructure that can improve the readiness, response, and monitoring of the US public health authorities to future biological threats.

Using a large national dataset, this study demonstrates that nearly all patients did not have follow-up testing captured in the dataset according to the FDA’s test instructions. It is possible that this result may be attributable to the increased availability of at-home tests over time, and it justifies the further development of data systems that can track individual patient testing across modalities for additional study. Despite low adherence to testing and noncompliant timing of testing, serial testing successfully identified infections missed by earlier tests at significant rates, indicating the public health benefit of serial testing.

With the availability of at-home testing, a significant proportion of second- and third-test patients likely used at-home tests rather than going back to the pharmacy, reducing the rates of detection through serial testing compared with those observed in this study. There was significant variability in second-test and third-test adherence rates among distinct demographic groups. Individuals with symptoms and children had lower odds of both second- and third-test adherence; Asian individuals had higher odds of second-test adherence but lower odds of third-test adherence; and individuals in the West had lower odds of second-test adherence but higher odds of third-test adherence. When clinical factors were incorporated into the model, adherence was significantly higher among patients with recent COVID-19 contact, but lower among those who had COVID-19 within the past 90 days. The rate of testing positive through serial testing was 24.9% for patients with symptoms on the second test and 4.2% for patients without symptoms on the third test. Finally, adherence was greater at community pharmacy sites compared with chain pharmacies, and second-test adherence for all test takers declined steadily over time. Altogether, these findings emphasize the need for additional targeted public health strategies to improve adherence, particularly among groups, sites, and later time periods with lower rates, to improve overall disease detection and management in future outbreaks.

## Abbreviations

Ag-RDT: antigen-detection rapid diagnostic test
AOR: adjusted odds ratio
CDC: Centers for Disease Control and Prevention
FDA: Food and Drug Administration
HRSA: Health Resources and Services Administration
ICATT: Increasing Community Access to Testing, Treatment, and Response
NAAT: nucleic acid amplification test
RT-PCR: reverse transcriptase–polymerase chain reaction

## Data Availability

The datasets analyzed during this study are available in the One CDC Data Platform (1CDP). Due to the sensitivity of the public health data and privacy regulations, the datasets are not publicly available but may be requested from the CDC. Anonymized line-level data are available upon request to the corresponding author and subject to an approved data use agreement.
